# Estimation of Stature from Percutaneous Hand Length Among the Students of A Medical College

**Published:** 2018-06-30

**Authors:** Anup Pandeya, Alok Atreya

**Affiliations:** 1 Department of Anatomy, Devdaha Medical College and Research Institute, Devdaha, Rupandehi, Nepal; 2 Department of Forensic Medicine, Devdaha Medical College and Research Institute, Devdaha, Rupandehi, Nepal

**Keywords:** *hand length*, *stature*, *students*

## Abstract

**Introduction:**

Anthropometry is commonly used technique for the measurement of bone and soft parts in Anatomy and other fields of science. It has practical implications mainly in the field of anatomy and forensic medicine. The present study is aimed to determine the average hand length with stature among the students of a medical college.

**Methods:**

A cross sectional study was done among 185 students including 98 females and 87 males of Devdaha Medical College and Research Institute. Hand length and stature were measured and collected data was entered and analyzed in SPSS 21. The differences in measurements of hand bone length and stature among males and females were calculated. The regression equation for the estimation of stature from hand length was calculated.

**Results:**

The mean hand length among the total population was 17.80+1.04 cm and stature was 160.50+6.73 cm. The mean hand length and stature were higher among males as compared to females. The range of hand length and stature among the total population is 16.00–21.20 and 146.00176.50 respectively. The regression equation for the estimation of stature from hand length revealed statistically significant value among the males and females.

**Conclusions:**

Our study reveals higher value of hand length and stature for males as compared to females. Also there is significant correlation between the hand length and the stature. The present values are obtained from the small population of an institute. Further studies have to be carried out to develop the regression formula for the Nepalese population with larger sample size.

## INTRODUCTION

Stature is regarded as one of the important factor to denote various parameters of a population including nutrition, health and genetics. Identification is the crucial part in forensic medicine and estimation of race, sex, age and stature is considered as the ‘big fours’ in forensic anthropology.^1^ The estimation of stature is based on two major methods: the anatomical method, which requires the complete skeleton, or the mathematical method, which employs regression formulae to estimate the stature.^1,2^ Body proportions and the dimensions of different body segments, including the vertebral column, long bones of the limbs and the bones of the hand and foot have been commonly used for stature estimation in different countries.^3,4,5,6^ There may be unavailability of complete skeleton or complete long bones when the bodies are dismembered or mutilated in wars, mass disasters, and crimes. Therefore, a practical alternative is to develop new standards formula that helps in the identification of skeletal remains.^7^

## METHODS

A cross-sectional study was done among the students of Devdaha Medical College and Devdaha College of Science and Technology from December 2017 to June 2018. The ethical approval was taken from ethical committee of Devdaha Medical College and Research Institute prior to the study. There were 185 participants who participated in the study including 87 males and 98 females. The participants were informed about the study protocols and personal identifier was removed before the data collection. The study participants were asked to stand in the anatomical position with bare foot and the height was measured with the help of stadiometer. The hand length was measured by using Vernier callipers. The variables were recorded as age, sex, hand length and height.

The hand lengths were measured in centimetres (cm) from the transverse crease of the wrist to the distal end of middle finger, which denotes the longest length of the hand.

The collected data was then entered and analyzed by using SPSS 21. The descriptive analysis was performed for frequency, mean and standard deviation (SD). Regression equations for the estimation of stature from hand length were calculated among the study participants. The inclusion criteria were: a) those participants who had no past history of hand bone fracture; b) those who had no congenital deformity of hand; and c) those who consented for the study. The exclusion criteria were: a) those having past history of hand bone fracture; b) those having congenital deformity of hand; and c) those participants who did not consent to participate.

## RESULTS

The present study comprised of 185 students in a medical college. Among them 85 (47%) were males and 98 (53%) were females. The mean value of hand length and stature were higher in males as compared to females. Similarly, the range of hand length and stature is far higher in males with the mean (18.58±0.84) and (165.941 ±4.30) respectively (Table 1).

**Table 1.** Mean and S.D. for hand bone length and stature among male and female students.

Gender	Total n (%)	Hand length			Stature
		Mean±SD	Range	Mean±SD	Range
Male	87 (47)	18.58±0.84	16.40–21.20	165.941 ±4.30	154.50–176.50
Female	98 (53)	17.10±0.62	16.00–18.60	155.68±4.40	146.00–169.00
Combined	185 (100)	17.80±1.04	16.00–21.20	160.50±6.73	146.00–176.50

The regression formula for the estimation of stature from hand length is

Height (y) = Constant + Independent variable x Hand length (x).

**Figure 1.**
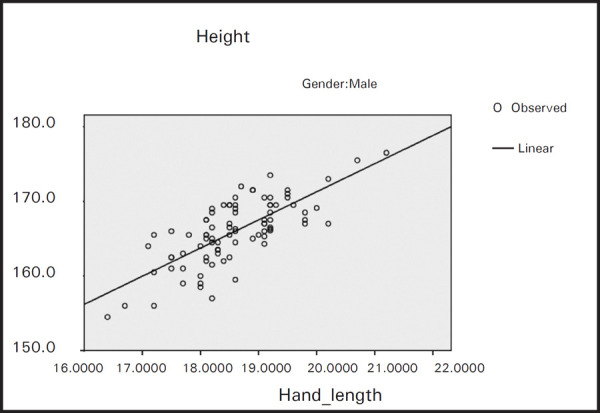
Regression of height on hand length for males.

**Figure 2.**
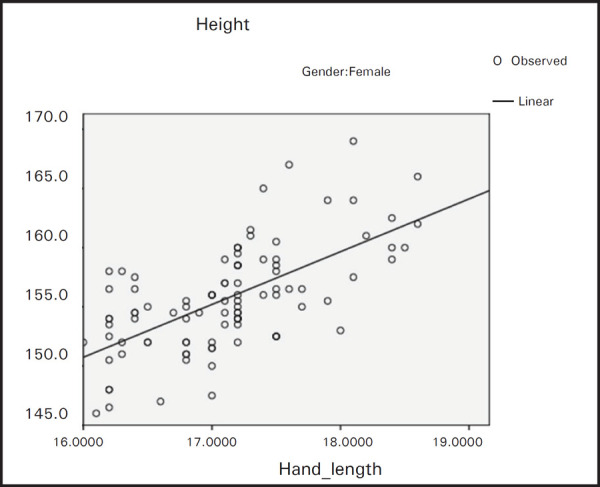
Regression of height on hand length for females.

**Figure 3.**
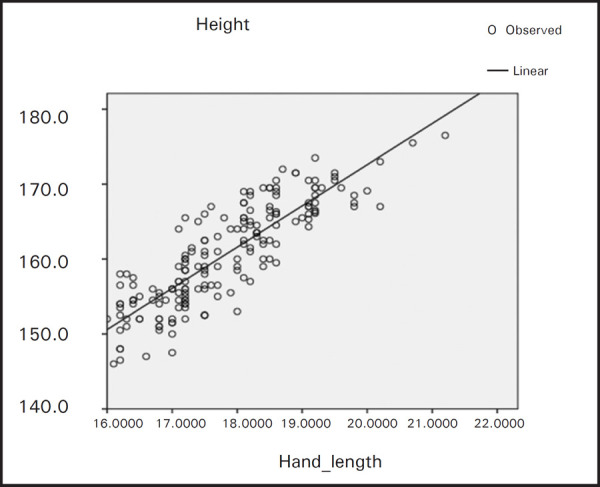
Regression of height on hand length for combined population.

From the formula we can estimate the stature from hand length of males, females and combined population (Table 2, Figure 1, 2 and 3).

**Table 2.** Regression equations for the estimation of stature from hand length.

Gender	Regression equation	Standard error	P
Male	95.86+3.76 × hand length	0.372	<0.001
Female	79.41 +4.46 × hand length	0.556	<0.001
Combined	62.59+5.50 × hand length	0.250	<0.001

689

## DISCUSSION

Personal identification is essential in medico-legal works. In cases of mass disaster where the body parts are dismembered, it is the role of forensic expert to identify the body and hand it over to the relatives for final rituals. Of this various methods of personal identification, estimation of stature from the measurement of body parts is widely used by forensic anthropologist.^8^ The estimation of stature from hand length and the derivation of regression equation was done in Punjabi males.^9^ The study performed in

Jos medical school of Nigeria among male students shows significant correlation between the hand length and stature which is similar to our study.^10^ Similar study conducted in Egypt also reported the positive correlation between the hand length and stature among the students of different colleges.^11^ Furthermore, the study conducted in different colleges of New Delhi also reported significant correlation between hand length and stature which is similar with our study.^12^ Similarly, the studies conducted in National Institute of Technology, Korea and Sudanese adults revealed statistically significant differences in the measurements of males and females with the higher values in males which is in agreement with our study.^13^ This may be the fact that the males have genetically larger size as compared to females. The puberty in females occurs about two years earlier as compared to the males which cause earlier fusion of epiphysis and there will be less time for growth.^11,14^ However, several studies reported that proximal limb bones are the better marker of prediction of stature than distal limb bones.^15,16^ But this was not in agreement with the study done in Sudanese adults and the study on done in Turkish males, where forearm length was better predictor for estimation of stature than upper arm length.^17,18^ Although the study helped us to calculate the regression equation among the male and female students of a medical college. Further studies are required in large population in various states of the country to build national statistics for Nepalese population.

## CONCLUSIONS

Our study reveals higher value of hand length and stature for males as compared to females. There is significant correlation between the hand length and the stature. The present values are obtained from the small population of an institute. Further studies have to be carried out to develop the regression formula for the Nepalese population with larger sample size.
